# Changes in Mice Brain Spontaneous Electrical Activity during Cortical Spreading Depression due to Mobile Phone Radiation

**Published:** 2008-06

**Authors:** Samera M. Sallam, Ehab I. Mohamed, Abdel-Fattah B. Dawood

**Affiliations:** 1 *Department of Physics, Faculty of Science, University of Benha, Benha, Egypt;*; 2 *Department of Medical Biophysics, Medical Research Institute, University of Alexandria, Alexandria, Egypt*

**Keywords:** EEG spontaneous activity, cortical spreading depression, mice brain, mobile phone, microwave radiation, slow potential changes

## Abstract

The objective of the present study was to investigate changes in spontaneous EEG activity during cortical spreading depression (CSD) in mice brain. The cortical region of anaesthetized mice were exposed to the electromagnetic fields (EMFs) emitted from a mobile phone (MP, 935.2-960.2 MHz, 41.8 mW/cm^2^). The effect of EMFs on EEG was investigated before and after exposure to different stimuli (MP, 2% KCl, and MP & 2% KCl). The records of brain spontaneous EEG activity, slow potential changes (SPC), and spindle shaped firings were obtained through an interfaced computer. The results showed increases in the amplitude of evoked spindles by about 87%, 17%, and 226% for MP, 2% KCl, and MP & 2% KCl; respectively, as compared to values for the control group. These results showed that the evoked spindle is a more sensitive indicator of the effect of exposure to EMFs from MP.

## INTRODUCTION

Studying the biological effects of microwave radiations, which are pulsed high-frequency electromagnetic fields (EMFs), is of great health concern, since there is an increasing debate regarding their implication in a number of public health problems (e.g., headaches, insomnia, and various cancers) (1-3). During their normal use, mobile phones (MP) emit EMFs, which are absorbed into the head and the brain of a user thus, altering its function (4, 5). Moreover, it has been shown that the type of tissue that absorbs the EMFs (e.g., head or chest) determins the observed changes in an electroencephalogram (EEG) (4). It has been shown also recently that the EMFs emitted by a commercial MP induce a local decrease in regional cerebral blood flow beneath the antenna in the inferior temporal cortex and an increase more distantly in the prefrontal cortex, consistent with the assumption that EMFs induce changes in neuronal activity (6).

In a recent study by our group, we have also shown that EMFs emitted by MP can elicit cortical spreading depression (CSD) in rat brains (7). Most typical EMFs-induced EEG changes are abnormal slowing and increases in the amplitude of EEG waves (8). Several reports have investigated the effect of EMFs from MP on slow potential changes (SPC) and spike unit activity of cerebral cortex in experimental animal models and in humans (7, 9-11), yet the functional significance of induced EEG changes remains unclear. Moreover, the EEG and neural activity changes have never been studied during CSD for animals exposed to EMFs from MP, since most studies have concentrated on how EMFs affect the human body and particularly the brain and cognitive function (12-14). The objective of the present study is to evaluate changes in EEG spontaneous activity during CSD in mice brain exposed to EMFs from MP.

## MATERIAL AND METHODS

### Animals

The experiments were carried out on 40 adult albino mice, which were purchased from the Holding Company for Biological Products and Vaccine, Cairo - Egypt, with an average weight 30.00 ± 2.00 g. Animals were housed in separate plastic cages, which were kept in a shielded chamber to reduce the normal environmental EMFs under similar conditions of temperature, illumination, acoustic noise, and ventilation. All animals received the same diet during the study period. The experimental protocol and use of animals in the present study were in accordance with national and international legal requirements and institutional guidelines.

Animals were divided into four groups as follows: a control group (n=10) for recording spontaneous EEG activity without any modifier, a group (n=10) for eliciting CSD by 2% KCl, a group (n=20) for studying the characteristics of EEG activity during direct EMFs exposure from MP for an hour daily for 10 days, which were thereafter classified into two groups: the third group (n=10) for studying MP exposure effects alone; and the fourth group (n=10) for studying the simultaneous effects of MP exposure together with 2% KCl. Animal preparation, methods of skull-trephine openings, and Ag-AgCl electrodes were performed as detailed previously by our group (7).

### Brain Activity Measurements

**Figure 1:**
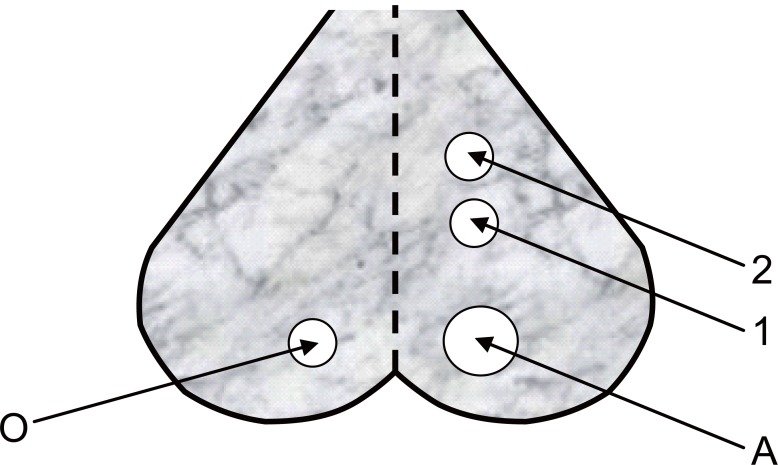
Schematic diagram for the experimental arrangement applied to all animals of the study where **A,** for the application of 2% KCl to cortical area; **1,** cortical electrode for electroencephalogram (EEG) recording; **2,** cortical electrode for slow potential change (SPC) recording; and **O,** reference electrode.

Recording of changes in spontaneous EEG activity and SPC during spreading depression (SD) were carried out according to the experimental arrangement shown in Figure 1. Mice were anesthetized by a subcutaneous injection with pentobarbital 40 mg/kg and the vital condition of the anesthetized animal was monitored by its normal breathing. Under anesthesia, the skin of the head was cut and the cerebral cortex of the right hemisphere was exposed by a 5 mm trephine opening over the occipital region (A) for the stimulation and two 3 mm openings over the parietal region (1 and 2) for the recording of EEG and SPC, respectively. A fourth trephine opening of 3 mm was made over the left hemisphere (O) for the reference electrode.

Wick Ag-AgCl electrodes were made to rest gently on the cortical surface avoiding any mechanical stress or damage. Ringer saline solution at room temperature (22°C) was used for washing the cortical surface from time to time to protect it from drying. The stimulation process was carried out using a 2% KCl solution applied through hole A (Figure 1) using a 2 mm piece of filter paper soaked by the solution for eliciting SD. Changes in EEG and SPC were recorded during SD caused by EMFs emitted from MP (935.2-960.2 MHz), which was kept in the silent mode during irradiation and placed near the cortical region of mice head. The EMF irradiation was carried out by directing the mobile antenna 1 cm apart over the occipital opening.

The SPC accompanying SD wave was recorded for anesthetized normothermic mice using the electrode in 2 (Figure 1), relative to the common reference electrode in O through a computer interface (PASCO 6500). Each experiment was repeated 6 times under the same conditions and arithmetic means and standard deviations for all measurements were calculated for further statistical analysis.

## RESULTS AND DISCUSSION

**Figure 2:**
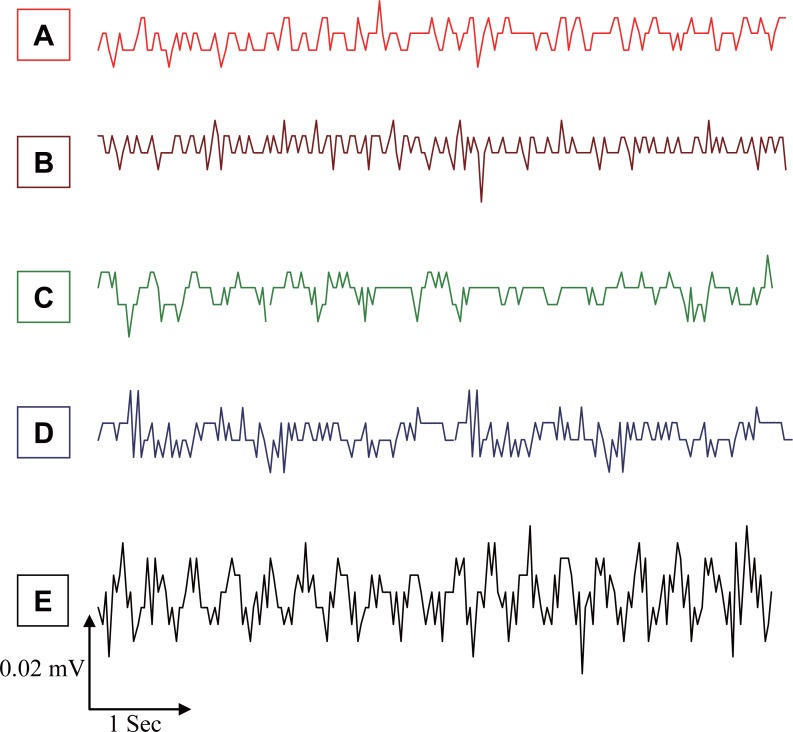
Typical sections of mice-EEG activity; **A,** Control; **B,** Pre-exposed; **C,** Exposed to MP; **D,** Exposed to 2% KCl, **E,** Exposed to MP & 2% KCl.

In the present work, we studied the effects of EMFs emitted from MP (935.2-960.2 MHz, 41.8 mW/cm^2^) on the spontaneous EEG activity, SPC, and evoked spindles of mice brain waves during SD. The changes in spontaneous EEG activity for different mice groups in Figure 2 are shown mainly as slow high-amplitude waves depending on the stimulus. The average changes in frequency (F, Hz) and amplitude (A, mA) of spontaneous EEG activity for different groups, as by Fourier Transform Analysis, are given in Table 1, which were significantly higher (*P*<0.01) by 115%, 58%, and 231% for MP, 2% KCl, and MP & 2% KCl, respectively, as compared to A values for the control group. The higher values of F and A of EEG activity due to EMFs from MP & 2% KCl are in line with the results of a recent study by Krause *et al*. (15), who showed that the rhythmic ongoing EEG activity is caused by minor currents of the neural activity, which reflects brain activity. Earlier studies have shown that brain exposure to low level microwave and radio–frequency EMFs were accompanied by structural deformation in the brain and alterations in the distinct aspects of brain EEG activity (16, 17). Moreover, the EMFs emitted by MP at 900 MHz affected vascular permeability in mice brain (18).

**Table 1:** Average changes in frequency (F) and amplitude (A) for brain spontaneous electroencephalogram (EEG) activity, Slow Potential Changes (SPC), and Evoked Spindles for the different study groups

Group	EEG Activity		Slow Potential Changes		Evoked Spindles	
	*F (Hz) ± 0.3 SD*	*A (mV) ± 0.2 SD*	*F (Hz) ± 0.3 SD*	*A (mV) ± 0.2 SD*	*F (Hz) ± 0.3 SD*	*A (mV) ± 0.2 SD*

**Control**	1.7669	0.0168	0.8877	0.0290	2.4359	0.4865
**Pre-exposed**	2.6162	0.0211	0.8183	0.0195	2.5325	0.6224
**Mobile Phone**	2.2941^a^	0.0362^a^	0.8310^b^	0.0206^b^	2.6522^b^	0.9100^a^
**2% KCl**	2.9735^a^	0.0265^a^	0.6155^a^	0.0238^b^	2.8880^a^	0.5714^b^
**Mobile Phone & 2% KCl**	3.1029^a^	0.0556^a^	0.7829^b^	0.0190^b^	3.1276^a^	1.5852^a^

Values are the average of 6 experiments and analysis were carried out using Fourier Transform Analysis.

a *P*<0.01,

b *P*<0.05 as compared to values for the Control group.

**Figure 3:**
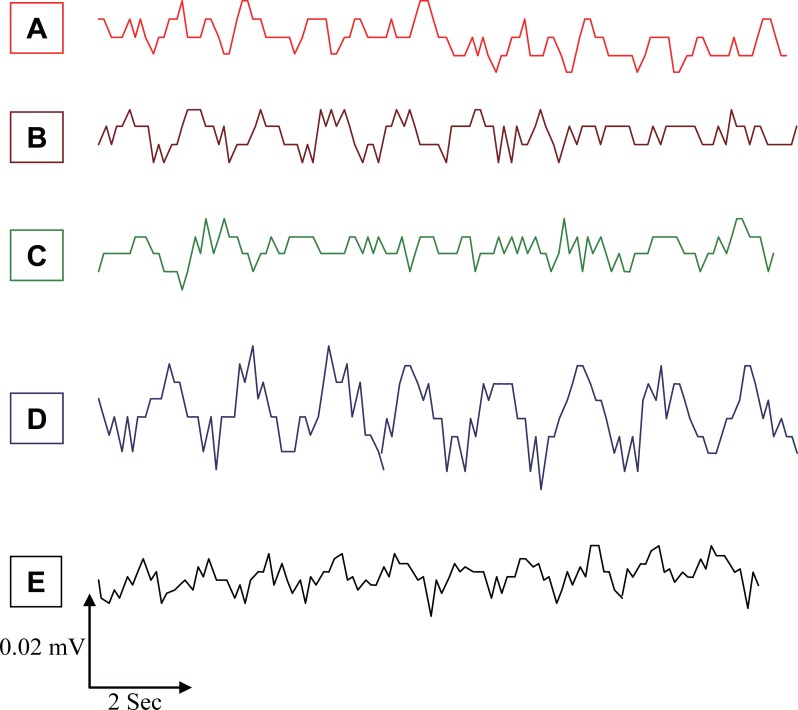
Typical sections of mice-EEG slow potential changes (SPC) for; **A,** Control; **B,** Pre-exposed; **C,** Exposed to MP; **D,** Exposed to 2% KCl; **E,** Exposed to MP & 2% KCl.

Although EMFs from a GSM MP (935.2 - 960.2 MHz, 8.5 mW) have been succeeded as an agent for evoking CSD in rats, Cleary *et al*. (19) reported that the flux of sodium and potassium ions across cell membrane can be affected by radio–frequency exposure over a wide range of frequencies. There was a pronounced decrease in slow EEG components for each stimulus, which resulted in the appearance of evoked SPC (Figure 3 and Table 1). The amplitude and pattern of the evoked potentials were largely dependent on the slow-wave phase from which they arose. The amplitude and duration of SPC values during SD waves for MP, 2% KCl, and MP & 2% KCl were significantly lower (*P*<0.05) than those for the control group by 29%, 18%, and 34%, respectively, which were in line with previous studies (7, 20-22).

**Figure 4:**
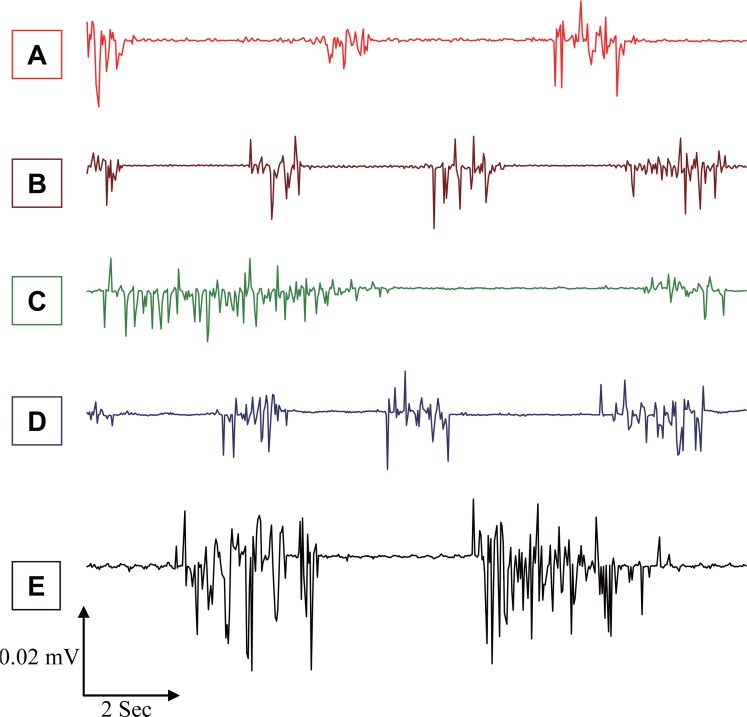
Typical sections of mice-EEG evoked spindles for; **A,** Control; **B,** Pre-exposed; **C,** Exposed to MP; **D,** Exposed to 2% KCl; **E,** Exposed to MP & 2% KCl.

Figure 4 and Table 1 show significant higher values of evoked spindles for F and A for the different stimuli, which were significantly higher (*P*<0.05) by 87%, 17%, and 226% for MP, 2% KCl, and MP & 2% KCl, respectively, as compared to A values for the control group. These significant high levels in spike numbers of evoked responses after EMFs exposure imply changes in the level of functional activity of the brain, since the functional state affects various electrophysiological parameters, particularly evoked responses (9). Thus, the main EEG reaction to microwave EMFs was a marked enhancement of slow waves and an increased number of spindle shaped firings. The increase in band-width of evoked spindles appeared particularly after exposure to EMFs from MP, which maybe due to the EEG reaction to EMFs exposure and the frequency of cortical neuron discharges. Similar changes of neuronal activity in rabbit cortex also have been observed after microwave exposure at power densities of 2.0-5.0 mW/cm^2^ (23).

The EEG is generated directly by cortical cell populations (24). EMFs with a radiation intensity of a usual GSM-telephone call may alter preparatory bioelectrical brain activity (25). Mobile communication may also influence other complex EEG features, such as the spectral hemispheric asymmetry (26), EEG coherence (27), or reinforcement of spontaneous EEG rhythms (28), and the EEG changes depend on the direct effect of EMFs on the cerebral cortex (29). However, for the whole brain the final configuration of this reaction is determined by interactions between the cortical and sub-cortical structures, since the burst of activity accompanying SD waves is probably a sign of the reticular activation projected to higher centers where it increases the firing rate of most cells (9).

Cortical SD increases threshold for elicitation of recruiting responses in the ipsilateral thalamus. The threshold for caudate spindles is also increased by ipsilateral cortical SD (20). Alteration of activity in different thalamic nuclei closely followed penetration of SD into functionally connected cortical region and the spindle-shape activity in the cortex is determined by direct and backward cortical-thalamic connections (30). We have previously found that MP radiation can elicit CSD (7), such radiation stimulates ipsilateral caudate nucleus and synchronizes evoked spindle originating from discharge frequency of neurons. The evoked spindle could be employed to differentiate between local and remote sources of EEG activity (20).

Despite controversies concerning the harmful biological effects of the use of GMS MP, numerous *in vitro* studies have shown that exposure to EMFs from MP can induce alterations in cell morphology and increase the expression of mitogenic signal transduction genes, cell growth inhibitors, and genes controlling apoptosis (14, 31-35). However, there is no definitive mechanism underlining the biological effects of EMFs from MP. Based on analyses of our data, we may suggest that the effect of cumulative exposure to microwave EMFs emitted from MP may affect apoptosis, but this needs extensive genetic studies. Finally, we conclude that the evoked spindle may be employed as a sensitive indicator of the effect of exposure to EMFs from MP rather than background activity. This suggestion agrees with a previous study on rabbits exposed to 12.5 cm microwave and reported that the evoked response is a more sensitive indicator of microwave effect (9).
